# Integrated Electronic Health Record System in a Tertiary Care Centre: A Single Centre Implementation Experience

**DOI:** 10.31729/jnma.7445

**Published:** 2022-04-30

**Authors:** Pawan Kumar Bajaj Agrawal, Baburam Giri, Priyanka Gupta, Rohit Khatri, Shreya Devkota

**Affiliations:** 1 Department of General Practice and Emergency Medicine, Amppipal Hospital, Palungtar, Gorkha, Nepal; 2 Department of Ear Nose and Throat- Head and Neck Surgery, Bluecross Hospital, Tripureshwor, Kathmandu, Nepal

**Keywords:** *electronic health record*, *hospital*, *Nepal*, *prescriptions*

## Abstract

An integrated electronic health record system is a common platform for efficient and active interaction of four major subsystems namely the medical record system, laboratory system, picture archiving and communication system and enterprise resource planning system. Barriers like knowledge and attitude of computer usage, time consumption, information technology workforce, electricity, cost of technology and devices, data safety and security deter institutions from implementation. However, implementation of this system is inevitable with its inherent advantages of efficient storage and timely retrieval, comparison of lab and imaging data over time, cash billing, institutional resource planning and finally ease of processing insurance claims as the Government of Nepal is looking forward to financing public health sector through the national health insurance system. Many hospitals struggle to implement and maintain integrated electronic health records. We aimed to discuss the steps undertaken to integrate this system in a district hospital setting with the inherent challenges and the subsequent impact.

## LOCATION

Amppipal Hospital is a 50 bedded rural government hospital in Gorkha with a catchment population of 22,676 and daily serves around 100 patients with five clinicians in the Out-patient Department (OPD) and around 20 patients in the In-patient Department (IPD). Almost 80 percent of the daily patients are insured with the government health insurance scheme. The institution provides medical care including life-saving surgeries. Approval was obtained from the hospital administration to access the necessary data for this article.

## SOFTWARE

The hospital management opted for an open-source project 'NepalEHR' (Nepal Electronic Health Record). It has been proven to have good acceptance among mid-level providers, the primary workforce in rural settings.^1-3^ The hospital had to pay only the implementation fee. NepalEHR has been largely customized to district hospital requirements in Nepal for a few years now. Most of the record-keeping from Health Management Information System (HMIS) and national programs like antenatal care, Tuberculosis (TB) and leprosy have already been incorporated.^4,5^

## PLANNING

The management coordinated with the technical team of NepalEHR for need assessment through online meetings over a two-month duration for need assessment, mainly focused on workflow establishment, a spectrum of clinical services available, knowledge of computer and technology, designing of local area networking and recommendation of procurement. The procurement consisted of 1 server, 1 data storage device, 10 desktops (each for registration, cashier, pharmacy, laboratory, 2 OPD rooms, 1 dental OPD, 1 physiotherapy station and IPD and visit summary station), 7 laptops (each for pharmacy, laboratory, 2 OPD rooms, eye OPD, minor procedure room and IPD), 3 printers (each for registration, IPD and visit summary station), local area networking cable, Local Area Network (LAN) ports for each device, two network switches and one wireless access point for inpatient department. The total implementation cost was estimated at around 2 million rupees.

## ON-SITE INTEGRATION

The technical team of three experts arrived on-site and established local area networking and also built a schema for future references. A dedicated server room with adequate security was established to decrease dependency on the internet. The continuous power supply was ensured for the server through the inverter. Two-hour orientation was provided to all the employees who were required to use EHR at their respective stations. The integration of EHR started from the registration station in the third week of January 2021 and progressed through lab, radiology, IPD and finally into OPD by the end of the week. Each user had a unique login password and every data in the patients' chart was saved with the time, date and name signature of the corresponding user. The daily workflow started with the registration of a client with the generation of a unique Identity Number (ID) which was used by the attending clinician to record patientinteraction including vitals, history and examination, and to order laboratory and imaging investigations. The respective departments carried out the investigations for individual ID and recorded it in EHR which is assessed by the clinician for decision making and a digital prescription was issued. The pharmacy dispensed as per the prescription in ID and processed for cash billing or insurance claims.

## FACILITATION

EHR required an uninterrupted power supply which was difficult in most of our settings. Despite electricity from grid lines, diesel generators and solar inverters, occasional interruption in EHR and hence the workflow was inevitable. The problem of interruption was however mitigated further with an optimal mix of laptops and desktops in respective stations. Monitoring was done to ensure a predetermined workflow and facilitate adoption by the employees. Regular feedback meetings were organized with the users to receive feedback on the necessity of new features in EHR. Consultation meetings were carried out with the NepalEHR technical team once a month to optimize the needs of users to facilitate adoption. Standard operating procedures were formulated to guide users. It was difficult to hire a technician for involved technology and networking in absence of adequate finance. So, the institution opted to train and evolve one of the existing employees with basic computer skills for networking and software management including basic customization.

## OUR EXPERIENCE

We compared the data during the fourth week of one month of integration and at the fourth week of nine months of integration. Writing a disposition was a must for all patients. Hence, completeness was considered when the clinicians properly recorded disposition notes. Errors in dosage form and dose, timings and the total amount of each drug required were considered prescription errors. OPD service time was considered as the time from registration to the time the clinician finally wrote the prescription and completed the consultation with disposition notes. The total number of patients registered in the fourth week of one and nine months were 476 and 496 respectively. Convenience sampling was employed for data from clinical stations. Since the disposition notes were not recorded for every patient, OPD service time was considered in 142 and 171 patients respectively each week. All the prescriptions in the given period, 456 at one month and 453 at nine months, were evaluated for errors. The number of patients attended by individual clinicians ranged from 12 to 39 at one month and 15 to 42 at nine months. The median time spent by the patients in the hospital for OPD service was 85 minutes with an interquartile range of 124 at one month and 85 minutes with an interquartile range of 130 at nine months. The OPD service time at one month, however, ranged from eight to 334 minutes. Similarly, at nine months it ranged from two to 407 minutes. Completeness of patients' sheets in EHR was observed at 30.04% at one month and 34.48% at nine months. Different studies had suggested that the completeness of EHR recording ranged from 35% to 99.4%.^6,7^ Fifty per cent of the digital prescriptions issued had some form of prescription errors at one month whereas it was reduced to 5.08% at nine months. A study reported a 16.10% prevalence of discrepancies in digital prescriptions.^8^ Thirty-one per cent of the patients in OPD received printed visit summaries which reduced to 4.20% at nine months. Government Health Management Information System (HMIS) reporting took one week at one month which was reduced to three days at nine months. Ten-day of OPD claims were pending for processing in the insurance management information system (IMIS) which improved to two days in nine months.

## WAY FORWARD

Our study outlined the challenges and solutions during the implementation of EHR in a low resource setting. Both integration and maintenance were difficult because of a shortage of technical human resources. Yet, the relentless usage and data obtained reveal the achievement of the implementation. EHR facilitated timely HMIS reporting and insurance claims processing. Adequate data recording improved over time but still, it was below par. Errors in recordings and prescriptions were inevitable with clicks and typing on computers but preventable with caution on the users' side. Customization of software was essential to ensure adoption and sustainability. A continuous effort from leadership and users to produce good quality data could be beneficial to both the institution and the patients.
